# Advances in Mid-Infrared Spectroscopy-Based Sensing Techniques for Exhaled Breath Diagnostics

**DOI:** 10.3390/molecules25092227

**Published:** 2020-05-09

**Authors:** Ramya Selvaraj, Nilesh J. Vasa, S. M. Shiva Nagendra, Boris Mizaikoff

**Affiliations:** 1 Department of Engineering Design, Indian Institute of Technology Madras, Chennai 600036, India; njvasa@iitm.ac.in; 2 Department of Civil Engineering, Indian Institute of Technology Madras, Chennai 600036, India; snagendra@iitm.ac.in; 3 Institute of Analytical and Bioanalytical Chemistry, Ulm University, 89081 Ulm, Germany; boris.mizaikoff@uni-ulm.de

**Keywords:** exhaled breath analysis, mid-infrared, MIR, non-invasive diagnostics, point-of-care (POC), infrared lasers, photoacoustic spectroscopy, quantum cascade lasers, QCL, biomarkers

## Abstract

Human exhaled breath consists of more than 3000 volatile organic compounds, many of which are relevant biomarkers for various diseases. Although gas chromatography has been the gold standard for volatile organic compound (VOC) detection in exhaled breath, recent developments in mid-infrared (MIR) laser spectroscopy have led to the promise of compact point-of-care (POC) optical instruments enabling even single breath diagnostics. In this review, we discuss the evolution of MIR sensing technologies with a special focus on photoacoustic spectroscopy, and its application in exhaled breath biomarker detection. While mid-infrared point-of-care instrumentation promises high sensitivity and inherent molecular selectivity, the lack of standardization of the various techniques has to be overcome for translating these techniques into more widespread real-time clinical use.

## 1. Introduction

**Figure 1:**
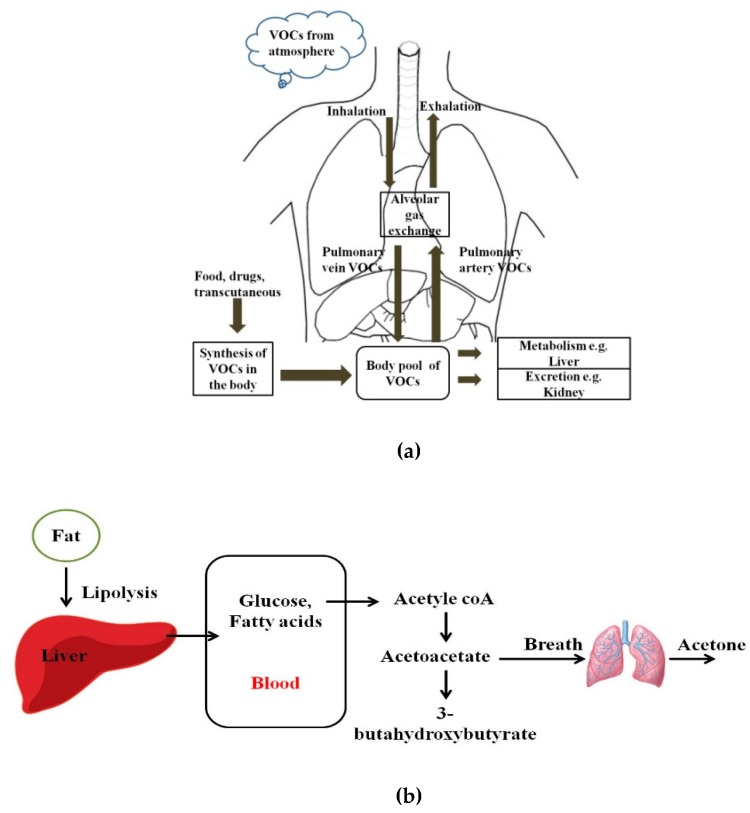
(**a**) Pathways for volatile organic compounds (VOCs) in the human body; (**b**) Schematic diagram of the formation of acetoacetate, beta-hydroxybutyrate, and acetone, which takes place in the mitochondrial matrix of the liver.

Human breath has always been a matrix of interest for disease diagnostics and monitoring owing to its inherently noninvasive access. Even in ancient times, human beings used to relate the odor of breath to diseases. Ancient Greek physicians assessed the aroma of human breath for disease diagnosis. Nebelthau (mid-1800s) identified acetone in the breath of diabetes patients, while Anstie (1874) isolated ethanol from the breath of alcoholics [1]. Linus Pauling’s work in the 1970s in breath analysis led to the detection of more than 200 volatile organic compounds (VOCs) in the exhaled human breath matrix, apart from then known compounds including CO_2_, O_2_, H_2_O, and N_2_ [2,3,4]. More recently, owing to advancements in detection and sensing technologies, researchers have found that more than 3000 VOCs may be present in exhaled human breath. However, trace quantities of gases, including VOCs, are also found anywhere in our immediate environments [5,6]. Figure 1 shows pathways for VOCs in the human body and the body pool of VOCs. VOCs are known to be present within the human body as a result of regular metabolic activity in the body, pathological disorders, and exposure to drugs. These endogenous VOCs are usually released into the bloodstream, and eventually metabolized or excreted from the human body by exhalation, skin emission, and urine [7]. On the basis of the analysis of breath samples collected from healthy and diseased human subjects during various studies, it was found that VOCs and their concentration in exhaled breath may act as biomarkers of selected diseases or pathophysiological conditions [8,9,10,11,12,13,14,15,16,17,18,19,20,21,22,23,24,25,26,27,28,29,30,31,32,33,34,35,36,37,38,39,40,41,42,43,44,45,46]. Despite the advantages of breath analysis as a non-invasive approach, the challenges with breath analysis remain in the variation in the concentration of target compounds from the subject. The variation or accurate quantification of the metabolic markers is influenced by gender, socio-economic and human demographic factors, use of medications, and dietary intake. Saturated hydrocarbons such as ethane, pentane, acetone, and aldehydes present in human exhale are an indication of lipid peroxidation of fatty acids [12,13]. For patients with diabetes, the body cannot synthesize insulin to break down glucose in the blood to provide energy. Therefore, the body undergoes lipolysis causing decarboxylation of acetoacetate, leading to the production of significantly increased concentrations of acetone in the breath, as shown in Figure 1b. Isoprene is formed along the mevalonic pathway in cholesterol synthesis [14]. Higher levels of breath pentane were also detected for cases of breast cancer, heart transplant rejection, myocardial infarction, schizophrenia, and rheumatoid arthritis [15,16,17,18,19,20].

Similarly, sulfur-containing compounds in breath are the result of incomplete metabolism of methionine in the transamination pathway. Moreover, nitrogen-containing compounds are at elevated levels in human breath for liver impairment or uremia. Nitric oxide has been identified as a biomarker of airway inflammation such as in asthma, allergic rhinitis, eosinophilic bronchitis, and chronic obstructive pulmonary disease (COPD) [21,22,23,24]. Carbon monoxide is indicative of an increase in oxidative stress or stimulation by pro-inflammatory cytokines, such as during smoking cessation [25,26].

The list of VOCs and inorganic compounds and their relation to potential disease states, as reported in various studies, is shown in Table 1. Breath analysis can reduce dependence on invasive diagnostics, such as bronchial biopsies and bronchoalveolar lavage for preliminary assessments. It can allow online examination with a shorter time for diagnosis compared with conventional analytical techniques, such as blood sample analysis. The research and analysis of VOCs in human breath gas considering the potential in assessment of environmental exposure to VOCs, as well as clinical diagnosis and disease state monitoring through evaluation of endogenous VOCs, have seen much interest in the past few decades. Numerous approaches have been reported for the assessment of VOCs in human breath. The exhaled breath (EB) matrix predominantly comprises CO_2_, H_2_O, O_2_, and N_2_ (% levels) next to much lower concentrated VOCs (ppm–ppt levels), which may mask the presence of other biomarkers for certain detection techniques. Hence, analysis techniques with pronounced sensitivity and selectivity are required for identifying and quantifying disease-related biomarkers against the highly variable breath matrix background.

Conventional breath analysis methods are usually based on offline sampling. Gas chromatography (GC) based separation coupled with mass spectrometry (MS) remains the ‘gold standard technique’ for trace gas analysis. Owing to its historical presence and analytical capabilities, GC–MS capitalizes on extensively developed libraries, which facilitate rapid compound identification at sensitivities down to ppt levels. However, these techniques involve manual sampling procedures and sample preparation [27,28,29,30,31]. Alternative technologies such as selected ion flow tube mass spectrometry (SIFT–MS), resonance enhanced multiphoton ionization mass spectrometry (REMPI–MS), and proton transfer reaction mass spectrometry (PTR–MS) provide rapid response times [32,33,34,35,36,37]. However, all these techniques involve very high instrumental costs, require highly trained personnel, and are laboratory-based tools. GC techniques coupled with the mass spectrometry are selective and sensitive, but cannot perform rapid trace-gas measurements, and precise calibration of the chromatographic column is required, which are not desirable for single-breath resolved breath analysis.

Alternative techniques like electronic nose sensors have found rapid developmental interest for their applications in small, affordable, point-of-care systems in breath analysis. Predominately, conducting polymer-based and metal–oxide semiconductor (MOS)-based arrays of sensors are used for determining multiple analytes. However, they are limited in molecular selectivity, high moisture sensitivity, and power demand [38].

Alternatively, ion mobility spectrometry (IMS) techniques such as those based on time-of-flight (TOFIMS) and differential ion mobility (DMS) have been reported for the detection of VOCs. DMS involves the separation of different ion species owing to differences in their ion mobilities in low and high electric fields. High-frequency asymmetric waveform field is used for the filtering of other species while selectively allowing ions of a particular species through the filter by tuning a low amplitude compensation field. DMS has resulted in a cost-effective, compact sensor without moving parts that allows real-time gas analysis [39,40,41]. However, owing to the low output current signal, the signal-to-noise ratio is low. Further, the sensitivity in measurements is also small and varies with the measurement species. For example, in the case of acetone and hexane, the sensitivity was estimated to be ≈120 pA per ppm (parts per million) and ≈700 pA/ppm, respectively [42].

Driven by the need for point-of-care (POC) medical instrumentation, laser spectroscopic-based noninvasive human exhale analysis has drawn increasing attention during the last decades since the development of new mid-infrared (MIR) laser sources [43,44,45]. POC instruments can be developed for monitoring of exhaled breath with high accuracy, sensitivity, detection limits, and reasonable prices. The major laser spectroscopic techniques under development for breath analysis include tunable diode laser absorption spectroscopy (TDLAS), cavity ring-down spectroscopy (CRDS), photoacoustic spectroscopy (PAS), cavity leak-out absorption spectroscopy (CALOS), hollow waveguide (HWG) absorption spectroscopy, and quartz-enhanced photoacoustic spectroscopy (QEPAS) [46,47,48,49,50,51,52,53,54,55,56,57,58,59,60,61,62,63,64,65,66,67,68,69,70].

**Figure 2:**
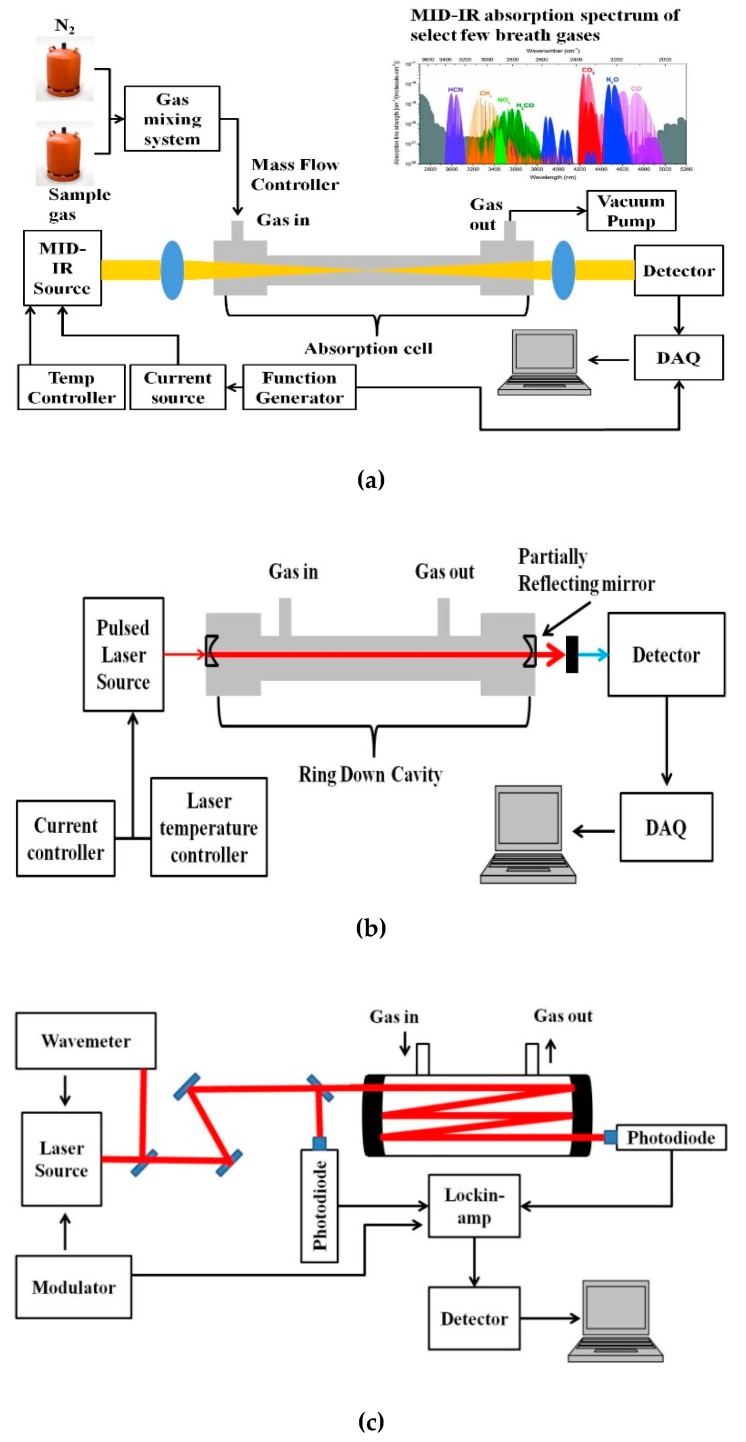
Simplified schematic of (**a**) mid-infrared (MIR) absorption spectroscopy, (**b**) cavity ring-down spectroscopy (CRDS), and (**c**) multi-pass spectroscopy (MUPASS). (DAQ: Data Acquisition system).

A basic schematic of the setup for MIR laser absorption spectroscopy is given in Figure 2a. The VOCs to be measured are usually in the ppm to ppb range, and hence the most sensitive techniques of absorption measurement have to be adopted for detection. Variations to the conventional laser spectroscopic method to improve the sensitivity of the sensor system include multi-pass spectroscopy (MUPASS) and CRDS, which are most widely used for such applications. Simplified schematics of such experimental systems are presented in Figure 2b,c. Their experimental setup and operation are detailed in [47,48,49,50,51].

Quantum cascade lasers and interband cascade lasers (QCLs, ICLs) in the MIR range have potential application, where many molecules relevant in exhaled breath diagnostics exhibit strong rovibrational absorptions are present. QCLs offer wide tunability and pronounced output power, leading to molecular selectivity, sensitivity, and improved signal-to-noise ratio for the detection of trace biomarkers in the exhaled breath matrix. Li et al. have used the ICL-based TDLAS approach for ethane detection in an exhaled breath at 3.34 µm, which is indicative of lung cancer and asthma. A detection limit of 1.2 ppbv was achieved at 4 s data acquisition time [52]. Ghorbani et al. have used an ICL-based TDLAS system for the identification of carbon monoxide (CO) in an exhaled breath at 4.69 µm using a multi-pass gas cell with a detection limit of 9 ± 5 ppbv at 0.07 s acquisition time, thereby resolving individual breath cycles (i.e., exhalation and inhalation profiles) [53]. Conventional spectroscopic methods like direct absorption spectroscopy are limited in sensitivity by the path length, as longer path length improves the sensitivity of the measurement. In such cases, integrated cavity output spectroscopy (ICOS), cavity-enhanced absorption spectroscopy (CEAS), and CRDS, allowing a sufficient path length of many kilometers with sensitivity as small as parts per billion or even parts per trillion, are used to improve the sensor system [54,55,56].

A variation of the CRDS technique is the CALOS, where, in contrast to the CRDS technique, a continuous wavelength (CW) source is used to tune one of the resonance modes of the cavity, allowing energy to build up inside the cavity [57,58,59,60]. At the time when the laser is turned off, the energy builds up, leaks out, and is detected by the photodetector. Murtz et al. developed this technique for the detection of ethylene (C_2_H_4_) using the spectral signature in the 10 µm band of the CO_2_ laser [57]. Later, Halmer et al. from the same group have demonstrated this technique for carbonyl sulfide (OCS) detection in breath samples with a detection limit of 438 ± 4.4 ppt. This group has also worked on ethane (C_2_H_6_) detection in breath samples in the 3.34 μm region. They compared the performance of CALOS with gas chromatography with flame ionization detection (GC–FID). Repeated tests concluded that the spectroscopy setup could detect ethane in less than 1 min, making it more suitable for continuous monitoring of patients, whereas chromatography required 30 to 60 min [58].

Though such techniques offer high sensitivity and can be adopted for continuous real-time monitoring of patients, such methods need excellent tuning of the cavity length. The optical cavity or multi-pass cell is also sensitive to mechanical vibration. Even the availability of either tunable light sources or high reflectance mirrors precisely for a particular wavelength of the target gas sample limits this technique from being widely used. Moreover, the need for high reflectance mirrors makes it relatively more expensive when compared with the other optical methods.

Recent advances in MIR waveguide technology have the potential to design advanced and compact instrumentation for trace gas analysis in this spectral regime. Hollow-core waveguides (HCW) in this region (3–20 µm) can facilitate the development of highly compact and sensitive trace gas sensing devices with potential usage in POC scenarios. Hollow-core photonic bandgap waveguides (HC-PBW) absorption spectroscopy for methane detection in the 3.4 µm region has been demonstrated by Nikodem et al. with sensitivities at the ppm-level [61]. For higher sensitivities, the length of the HC waveguide may be increased. However, bending the fiber for maintaining a compact footprint will cause optical losses.

**Figure 3:**
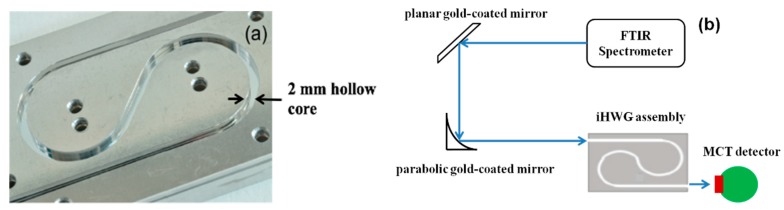
(**a**) Substrate-integrated hollow waveguide (iHWG) assembly with a yin-yang structure; (**b**) Schematic of the iHWG sensor system [62]. (FTIR: Fourier Transform Infrared Spectrometer; MCT: Mercury-Cadmium-Telluride).

Recently, a novel concept of substrate-integrated hollow waveguides has been introduced [62,63,64,65,66,67,68,69,70]. The substrate-integrated hollow waveguide (iHWG) is based on a layered structure with the light-guiding channels integrated into a rigid solid-state substrate material. The geometry of the iHWGs studied with a 2.0 mm hollow core edge length and a yin-yang structure is shown in Figure 3a. The experimental setup and the integration of the iHWG with the detector are shown in Figure 3b and explained in [62]. The significant advantage of the iHWG is that any low-cost substrate material combined with a cost-effective fabrication or replication technique, including hot embossing or even 3D printing of iHWGs [68], may enable a device fabrication strategy that is fundamentally different from conventional fiber optic HWG fabrication technology at a fraction of the cost. The analytical performance, that is, the energy throughput of iHWG, depends on the channel geometry and the surface roughness. Superior surface coatings are essential to reduce the reflection losses, and the iHWG channel length has to be specifically tailored for individual breath gases under study. However, the detection limits of this technique for breath gas analysis can be further improved when combined with other analytical methods.

Alternatively, hollow core waveguides have also been used in fibre enhanced Raman spectroscopy (FERS) [71,72]. FERS is based on spontaneous Raman scattering (SRS), which is an inelastic scattering process that can measure multiple gas species with a single laser at a fixed wavelength [73]. Hence, SRS has potential in exhaled breath analysis for its inherent ability to determine a large number of species. However, the major disadvantage of SRS in gaseous medium is the low intensity of the scattered signal, which can be overcome by signal amplification techniques like multi-pass optical cavities [74,75]. On the other hand, the application of HWGs serving as a miniaturized sample container can also improve the sensitivity by increasing the interaction of the propagating light with the molecules at low sample volumes. The detection limits, spectral characteristics, and laser techniques employed for significant biomarkers are shown in Table 2. PAS is another technique, where instead of a photodetector for laser absorption measurement, a microphone is used for acoustic signal measurement. This paper specifically discusses photoacoustic spectroscopy and its application to breath gas analysis. It also presents the challenges and perspectives of the suitability of PA-based spectroscopic methods for the development of point-of-care instruments for breath gas analysis.

## 2. Photoacoustic Spectroscopy for Breath Gas Analysis

**Figure 4:**
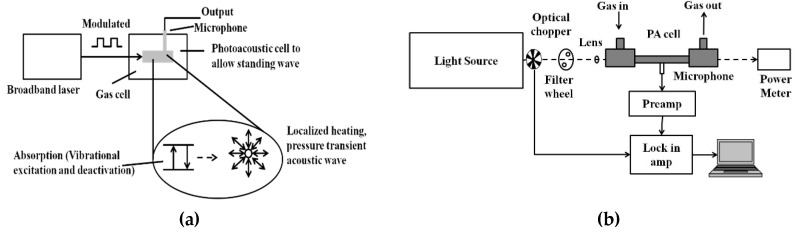
(**a**) General schematic of the photoacoustic (PA) signal generation process; (**b**) general schematic of the PA sensor architecture.

PAS is a zero-background technique and the PA signal is less affected by scattering. Hence, it has been widely used for trace gas detection at the part per billion or even part per trillion levels. Figure 4a shows the schematic of the PA signal generation process.

In photoacoustic spectroscopy, as the name suggests, light energy is converted into sound energy in a series of steps. The modulated or pulsed light is focused into a gas cell containing the target sample to be analyzed. If the frequency of the light source matches with the vibrational frequency of the gas molecule to be analyzed, the light is absorbed. Periodic amplitude modulation in the light source causes a periodic acoustic vibration in the gas cell. This acoustic vibration is detected by the microphone and converted into an electrical signal [76,77,78,79,80]. The general schematic for the PAS technique is shown in Figure 4b. Mid-IR light sources like QCL and optical parametric oscillator (OPO) are widely preferred for their narrow linewidths and tunability.

**Figure 5:**
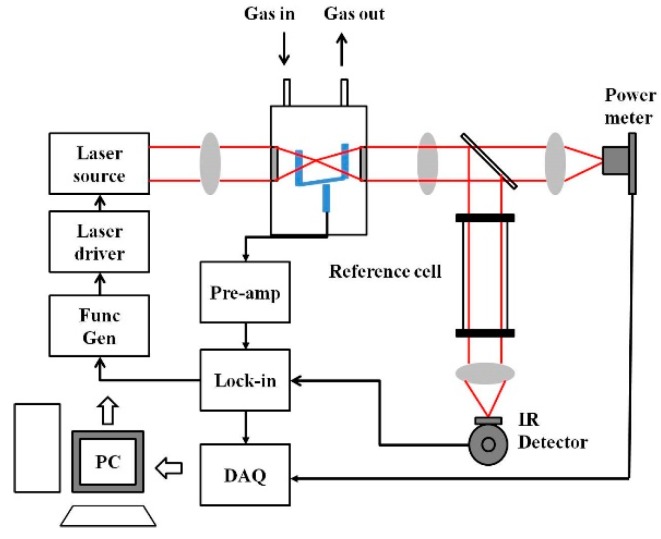
General quartz-enhanced photoacoustic spectroscopy (QEPAS) sensor architecture.

On the other hand, broadband sources matching with broadband spectra of species of interest can be used, which allows low power based multiple-gas sensing. Recently, QEPAS based on quartz tuning forks (QTF) as a sound transducer for the PAS technique has been increasingly used for selective and sensitive sensing [81,82,83,84,85,86,87,88,89,90,91,92]. The general architecture of a QEPAS system is shown in Figure 5. Commercially available QTFs are tiny (4 mm × 1.5 mm × 0.35 mm), and hence allow small sampling volumes.

### 2.1. Selected Breath Biomarkers Detected by Photoacoustic Techniques

#### 2.1.1. Ammonia (NH_3_)

Many studies have shown that ammonia in exhaled breath can be used for detecting chronic kidney disease (CKD), because, in patients with CKD, the accumulated urea cannot be excreted by the kidneys, but is degraded by the salivary urease into ammonia, which is exhaled through the breath. A variety of medical conditions, including liver and kidney disorders [93,94], as well as helicobacter pylori infections [95], can be detected by exhaled ammonia concentration. Narasimhan et al. have used a tunable line switched CO_2_ laser operating in the 9 and 10 µm wavelength range. They were able to detect 100 ppb ammonia-detection sensitivity [96]. Lewicki et al. employed an EC-QCL centered at 10.2 um with an output power of 42 mW using the QEPAS technique for ammonia measurement in human breath. The detection sensitivity for exhaled ammonia is at <10 ppb level with 1 s time resolution [97]. On the other hand, Bakhirkin et al. used a CW mid-infrared DFB quantum cascade laser centered at 10 µm, an output power of 30 mW, and quartz-enhanced photoacoustic spectroscopy for ammonia detection with a detection sensitivity of 20 ppbv (1σ) at a 0.3 s time resolution [98].

#### 2.1.2. Ethane (C_2_H_6_)

Lipid peroxidation that is the reaction between omega-3 fatty acids and reactive oxygen species releases ethane in the human body, which can be identified in the human breath. Ethane has also been noted as an indicator of oxidative stress, which in turn has been said to play an essential role in the pathophysiology of several common diseases. Studies show that cancer, cardiac disease neurodegenerative disease, psychiatric illness, stroke, and diabetes patients have high oxidative stress, and hence it has been proposed to have any involvement in the initiation of disease such as in cancer [99,100]. Moreover, studies on patients with disorders such as attention deficit hyperactivity (ADH), schizophrenia, asthma, and hypertension during pregnancy show a common underlying factor of increased oxidative stress and ethane in the exhaled breath [101,102,103]. However, it is unclear as to whether systemic lipid peroxidation changes ethane concentrations or whether they are mainly pulmonary in origin, because, in addition to pulmonary diseases, smoking tobacco has been said to elevate ethane concentrations [104,105]. Herpen et al. demonstrate a detection limit of 0.01 ppb with a high power (1.2 W) OPO (tuning range 3–3.8 µm) that is continuously tuned over 24 GHz during the trace gas measurements [106]. Ethane concentrations in healthy humans range between 0 and 12 ppb, hence low ppb detection limits are required for such breath gas measurements.

#### 2.1.3. Ethylene (C_2_H_4_)

Ethylene, as a biomarker, has been studied in dialysis patients. Studies show a relation between oxidative stress and fatality in patients with renal failure, especially elderly patients. A conventional PAS sensor using a CW carbon dioxide laser (10.53 µm) was developed to monitor C_2_H_4_ concentration in elderly patients (age 70–80 years) [107]. The ethylene concentration was found to vary between 0.15 ppm and 0.8 ppm before and after Hemodialysis, respectively. The healthy concentration level was found to be ~0.007 ppm. QEPAS-based C_2_H_4_ sensors were reported previously using a 3.32 µm DFB laser (1.5 mW) to achieve a minimum detection limit of 63 ppm at 25 s averaging time [108]. Wang et al. used QEPAS-based C_2_H_4_ sensor by exploiting the C_2_H_4_ spectra near 10.5 µm. The CW DFB QCL with ~23 mW power output at the target wavelength near 10.5 µm achieved a minimum detection limit of 50 parts per billion (ppb) at an averaging time of 70 s [109].

#### 2.1.4. Acetone (CO(CH_3_)_2_)

For patients with diabetes, the body cannot synthesize insulin to break down glucose in the blood to provide energy. Therefore, the body undergoes lipolysis, causing decarboxylation of acetoacetate, leading to the production of significantly increased concentrations of acetone in the breath [110,111]. Hence, the breath of diabetic patients is characterized by the fruity odor of acetone. Tyas et al. have used a CO_2_ laser in the 10.6 um range for the detection of acetone. A group of healthy individuals was studied against a group of patients with type 2 diabetes mellitus (DM). In the group with type 2 DM, the acetone range was to vary between 101 and 162 ppb, while in healthy individuals, the acetone range was between 15 and 85 ppb [112].

#### 2.1.5. Nitric Oxide (NO)

Nitric oxide is an important biomarker for chronic obstructive pulmonary disease (COPD). The patients often exhibit symptoms that alter their performance statuses such as productive cough, worsening dyspnea, peripheral muscle weakness, and nutritional abnormalities. Measurement of exhaled nitric oxide (eNO) is a non-invasive method of assessing airway inflammation. In patients with COPD, the peripheral airway (bronchioles) is the leading site of obstruction and inflammation [113,114], and the peripheral nitric oxide levels may be more predictive of the disease course and control. Tittel et al. have designed a 2f wavelength-modulation spectroscopy-based QEPAS detection approach for NO monitoring in COPD patients. They utilized an EC-QCL source operating at the NO R (6.5) absorption doublet centered at 1900.08 cm^−1^ (λ ~ 5.263 μm). The minimum detection limit achievable is ~5 ppbv with a 1 s data acquisition time [115]. The estimated exhaled breath nitric oxide concentrations are between 0 and 100 ppb in healthy humans.

#### 2.1.6. Methane (CH_4_)

Methane as a breath gas is of considerable interest because it is considered as a potential biomarker for stomach inflammatory diseases and colorectal cancer. Various studies demonstrate that high lipid, low-fat diet, and elevated bile salts, as well as the presence of colonic anaerobic bacteria, are a source of methane in the intestine, which then traverse the intestinal mucosa and are absorbed into the systemic circulation. As it has low solubility, it is rapidly excreted by the lungs. Breath methane analysis has been shown in various studies for the diagnosis of carbohydrate malabsorption syndromes and small intestinal bacterial overgrowth and, if the exhaled methane is more than one ppm as compared with ambient levels, the patient is considered a methane producer. Bauer et al. presented the development of a Raman amplifier system operating at 1651 nm and its application for trace gas sensing with a miniaturized 3D printed PAS cell. The system exhibited high sensitivity towards methane sensing with the least detection limit of 17 ppb at a signal acquisition time of 130 s [116].

**Figure 6:**
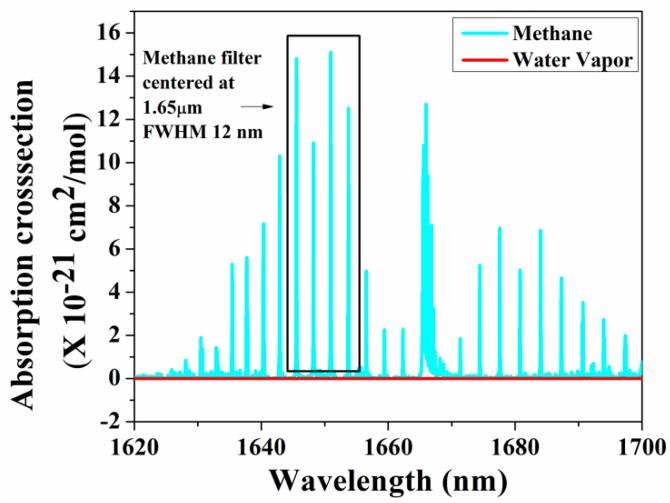
Broadband absorption spectra for methane.

As the amplitude of the photoacoustic signal is directly proportional to the input power and the absorption cross-section of the target gas, in recent years, many research groups have used QCL and ICL in the mid-infrared and THz regions, which are considered as the molecular fingerprint regimes. Furthermore, the QEPAS technique, along with a QTF, has been used to fabricate small, compact PAS systems [117]. Petersen et al. developed a QEPAS sensor using an OPO as a light source in the 3.1–3.7 µm region for detecting methane in exhaled breath. A minimum detection limit of 32 ppbv at 190 s integration time was achieved [118]. A broadband photoacoustic technique for CH_4_ detection in the 1.65 µm region was developed, where multiple absorption lines of methane were utilized to produce the photoacoustic signal. A bandpass filter with a full width at half maximum (FWHM) of 12 nm was chosen with a centre wavelength at 1.65 µm where the interference owing to moisture is minimal, as shown in Figure 6 [119]. A multi-wavelength algorithm can be used to estimate the cumulative PA signal amplitude analytically and a least detection limit of 0.05 ppm was reported [120].

## 3. Challenges and Perspectives

While the potential advantages of breath analysis are apparent, the variability of breath measurements has been quite high, and thus their potential has not been fully utilized to date. Though chromatography is considered the gold standard in breath gas analysis, real-time monitoring is not feasible via such laboratory-based techniques probing discrete samples. On the other hand, laser spectroscopic methods can be applied to real-time monitoring of breath gases. With the advancement in MIR lasers, compact handheld devices can be established with high sensitivity, molecular selectivity, and reproducibility. Even though such laser spectroscopic techniques offer sensitivities in the ppb to ppt concentration range, there are still several improvements required to render MIR spectroscopy a useful clinical tool in routine breath gas monitoring. This an issue for all sensing techniques, as MIR spectroscopies need to deal with the uncertainty on how the presence and/or up/down regulation of selected biomarkers may be mapped onto specific diseases, disease progression, or therapeutic interventions. For example, ethane can be indicative of cancer, cardiac disease neurodegenerative disease, psychiatric illness, stroke, and diabetes. Hence, the pathobiology of the breath compounds has to be extensively studied for establishing diagnostically relevant and usable correlations between biomarkers and the disease. As several studies have shown that, indeed, multiple rather than individual VOCs giving rise to pattern changes may be used to more reliably associate with a particular condition, MIR diagnostics are certainly at the forefront of multi-component sensing techniques utilizing fingerprint patters for detecting several VOCs. Appropriate selection of broadly tunable QCL, ICL, OPO, or supercontinuum light sources enables addressing such complex fingerprints, and may aid in further lowering detection limits, especially in broadband PAS measurements. In conclusion, even though more work certainly needs to be done on MIR sensing technologies as well as on establishing reliable pathobiologically relevant breath biomarker panels, appropriate MIR sensing systems may differentiate between healthy and diseased individuals in a statistically sound fashion appear to be on the horizon.

## Figures and Tables

**Table 1:** List of biomarkers and their relation to potential disease states.

S. No	Disease	Potential Source of Biomarker	Biomarker	Concentration (diseased)	Concentration (healthy)	Reference
1	Diabetes	Oxidation of non-osterified fatty acids	Acetone (CH_3_)_2_CO	T1D: >2.2 ppm, typically >10 ppm	0.39 to 0.85 ppm	[1,31]
2	Colorectal cancer	(1) High lipid, low-fat diet; (2) increased presence of bile salts; (3) presence of colonic anaerobic bacteria	Methane (CH_4_)	8 to 50 ppm	3 to 8 ppm	[8]
3	Non-small cell lung cancer (NSCLC)	Oxidative stress	Interleukin-6	9.3 to 9.9 pg/mL	3.3 to 3.7 pg/mL	[11]
4	Blood cholesterol	Mevalonate pathway of lipid (cholesterol) metabolism	Isoprene	-	3.5 to 10.5 nmol/L.	[14]
5	Myocardial infarction	Lipid peroxidation, leading to the pathogenesis of tissue damage	Pentane (C_5_H_12_)	-	0.3 to 0.8 nmol/L	[18,19,20,32]
6	Obstructive sleep apnea	Oxidative stress	Interleukin-6 (IL-6), 8-isoprostane	8.4 to 9.0 pg/mL 6.7 to 7.1 pg/mL	1.5 to 1.7 pg/mL 4 to 5 pg/mL	[10]
7	Smoking		Carbon Monoxide (CO)	2 to 20 ppm (smokers)	0.4 to 0.8 ppm (non-smokers)	[25,26]
8	Renal failure, oral cavity disease	Lipid peroxidation	Ammonia (NH_3_)		0.25 to 2.9 ppm	[9,37]
9	Scleroderma, cystic fibrosis		Ethane (C_2_H_6_)		0 to 12 ppb	[8,13]
10	Asthma, acute lung injury, inflammatory lung diseases, lung infection, lung cancer, rhinitis	Nitric oxide synthase	Nitric Oxide (NO)		<35 ppb	[21,22,23]

**Table 2:** Spectral fingerprints, laser techniques employed, and detection limit of select few biomarkers.

S. No	Biomarker	Technique	Light Source	Wavelength (µm)	Detection Limit	Reference
1	Nitric Oxide (NO)	CEAS	QCL	5.262961	5 ppb	[45]
		ICOS	QCL	5.22	0.4 ppb	[46]
		CALOS	CO laser	5	7 ppt	[47]
		TDLAS	IV–VI laser	5.2	1.5 ppb	[48]
		MP absorption spectroscopy	QCL	5.2630	0.3 ppb	[49]
		CEAS	QCL	5.2630	30 ppb	[50]
2	Carbonyl Sulphide (OCS)	CALOS	CO laser	5	438 ± 4.4 ppt	[51]
		CEAS	QCL	4.8716	0.9 ppb	[50]
3	Ethane (C_2_H_6_)	CALOS	ECDL 800 nm an d Nd-YAG 1064 nm with PPLN	3.34	1–100 ppb	[52]
		CEAS	Tunable laser system	3.3481	0.3 ppb	[50]
		TDLAS	ICL	3.34	1.2 ppb	[53]
4	Methane (CH_4_)	HCF		3.4	ppm	[54]
		MP absorption spectroscopy	QCL	7.874	1 ppb	[49]
5	Acetone (CO(CH_3_)_2_)	WMS	DFB-ICL	3.367	0.58 ppm (1 s) 0.12 ppm (60 s)	[55]
		WMS-MP-Broadband DAS	EC-QCL	~7.4	15 ppbv (<10 s)	[56]
		CEAS	QCL	8.22	0.51 ppm	[57]
6	Ammonia (NH_3_)	MP absorption spectroscopy	QCL	10.341	0.2 ppb	[49]
		WMS-MP	QCL	9.062	7 ppbv	[58]
		Pulsed CRDS	QCL	10.309	50 ppb	[59]
7	Carbon Monoxide (CO)	TDLAS-MP	ICL	4.69	9 ± 5 ppbv	[60]
8	Ethylene (C_2_H_4_)	MP absorption spectroscopy	QCL	10.416	0.5 ppb	[49]
		CALOS	CO_2_ laser	10		[61]
9	Formaldehyde (HCHO)	MP absorption spectroscopy	QCL	5.665	0.15 ppb	[49]

CEAS: cavity-enhanced absorption spectroscopy, ICOS: integrated cavity output spectroscopy, CALOS: cavity leak-out absorption spectroscopy, TDLAS: tunable diode laser absorption spectroscopy, MP: multipass, WMS: wavelength modulation spectroscopy, DAS: direct absorption spectroscopy, CRDS: cavity ring-down spectroscopy, QCL: quantum cascade laser, ECDL: external cavity diode laser, DFB-ICL: distributed feedback-intracavity laser, HCF: hollow core fibre, PPLN: periodically poled lithium niobate.
